# Meaningful Associations Redux: Quantifying and interpreting effect size in the context of the Adolescent Brain and Cognitive Development study^☆^

**DOI:** 10.1016/j.dcn.2025.101630

**Published:** 2025-10-10

**Authors:** Anthony Steven Dick, Jonathan S. Comer, Mohammadreza Bayat, Marilyn Curtis, Timothy Hayes, Shannon M. Pruden, Samuel W. Hawes, Raul Gonzalez, Angela R. Laird, Paulo A. Graziano

**Affiliations:** a Department of Psychology, Florida International University, Miami, FL, 33199, USA; b Department of Physics, Florida International University, Miami, FL, 33199, USA

**Keywords:** ABCD study, Effect size, SESOI, Equivalence testing, Estimation, Big data

## Abstract

The Adolescent Brain Cognitive Development (ABCD) Study represents a pioneering initiative that aims to unravel the complexities of behavioral and neural development in youth. In this paper, we address the challenges inherent in extracting meaningful insights from the extensive data compiled by the ABCD initiative. Our focus is on advocating for best practices in reproducible research, interpretation of effect size, and reporting of principled results. Central to this discourse is a detailed examination of effect sizes within the expansive ABCD dataset, and how they can be meaningfully interpreted in the context of large-scale research. We describe the hurdles associated with transitioning from conventional small-sample studies to the opportunities and challenges of large samples, including the phenomenon of statistically significant but practically trivial effects. To promote transparent and rigorous inference, we present a four-part framework to evaluate observed effects: researchers should **define** a smallest effect size of interest (SESOI), **compare** estimates to relevant benchmarks, **test** whether observed effects exceed meaningful thresholds (e.g., through equivalence testing), and **visualize** results to enhance interpretation and communication. Applying this framework yields a clearer, more cumulative understanding of effect size interpretation and contributes substantively to the refinement of scientific practices within adolescent brain and cognitive development research.

## 1 Meaningful Associations Redux

In our recent publication titled “Meaningful associations in the Adolescent Brain Cognitive Development Study” (Dick et al., 2021), we explored the objectives and framework of the Adolescent Brain Cognitive Development (ABCD) Study. That discussion raised a number of foundational issues, including the complexities of making population inferences, conducting hypothesis testing, ensuring statistical power and precision, managing covariates, and promoting best practices in reproducible research and statistical reporting. However, one central issue – the meaningful interpretation of effect sizes in large datasets – requires deeper examination. In this paper, we take up that challenge, focusing specifically on how to discern whether observed effects in the ABCD dataset are merely statistically significant or substantively meaningful.

Large-scale studies like ABCD enable researchers to detect even minuscule effects with high power. But this benefit also introduces interpretive complexity: when nearly all effects reach statistical significance, the critical question becomes whether those effects are practically important. Our goal is to encourage researchers using ABCD data to move beyond binary significance testing and toward an interpretive framework that centers on estimation, relevance, and precision.

To guide this transition, we propose a four-part framework for evaluating effect sizes: **define**, **compare**, **test**, and **visualize**. First, researchers should **define** a smallest effect size of interest (SESOI), based on theoretical, clinical, or practical considerations. Second, observed effects should be **compared** to appropriate benchmarks – such as prior literature, meta-analyses, or normative distributions – so they can be contextualized. Third, effects should be **tested** to determine whether they exceed (or fall below) meaningful thresholds, using tools such as confidence intervals or equivalence testing. Finally, researchers should **visualize** their results to aid interpretation, improve communication, and highlight patterns that are not apparent from statistical values alone.

In the following sections, we begin with a brief review of relevant statistical foundations, including the distinction between effect size and error variance. We then survey common effect size metrics appropriate for ABCD research and describe both the limitations of small-sample studies and the new challenges that arise with very large samples. We illustrate these issues using three examples from ABCD, each demonstrating the application of our framework to real-world data. Throughout, we emphasize the importance of interpreting results in light of clinical and practical significance, not merely statistical detection. We conclude with concrete recommendations based on the define–compare–test–visualize framework to support transparent, meaningful, and cumulative science in the era of big data.

## 2 “Meaningful effects” in the frequentist NHST tradition

In the frequentist null-hypothesis significance testing (NHST) tradition, there are two fundamental components reported in most statistical analyses: the statistical significance of the effect, and the size of the effect. In this tradition, researchers are typically interested in showing that the size of the effect is a value other than zero in the population of interest, and that the samples collected will evidence that with a high degree of reliability. For example, if we are interested in showing that the population average height of men is different than the population average height of women, we are trained to collect a random and representative sample of men and women and measure their heights. Here, we are assured to get a difference in heights, simply because the measurement tool (say, a medical scale with a height bar) has sufficient measurement resolution and reliability. It would be exceedingly odd if we found the averages of both of the samples to be the *exact same* to, say, a millimeter resolution. So the answer is already known before conducting the experiment: “The average heights are different” or more precisely “The average difference in heights is not zero.” (exactly zero is what Cohen, 1994 called “the nil hypothesis”). But we are also trained, following years of statistical advances (Stigler, 1986), to ask the follow-up question: “Are they *significantly* different?” To answer this question, we are taught to conduct a significance test.

### 2.1 P < .05 is not inherently meaningful

While misguided, in the minds of most researchers, the goal of this significance test is to find a statistically significant effect, and in the parlance of NHST, this means to find a *p* value below the prescribed α of (usually) 0.05. However, it is worth examining what this means with respect to the typical research question (Cohen, 1994). In simple terms, the *p* value is the “observed significance level” for the test hypothesis. In more complex terms, Wasserstein and Lazar (2016) define it as “the probability under a specified statistical model that a statistical summary of the data (e.g., the sample mean difference between two compared groups) would be equal to or more extreme than its observed value” (Wasserstein and Lazar, 2016). This is a nice technical definition, but it leaves most people more confused. A more accessible notion is that *p*-values indicate how incompatible the data are with a specified statistical model (including all its assumptions). Thus, a small *p* value flags the data as being unusual if all the assumptions, including the test hypothesis, were correct (Greenland et al., 2016). But it tells us nothing about whether the effect is large or small, whether it is meaningful in a practical sense, or whether it is “clinically meaningful” (see Section 4.2 below). Furthermore, the *p* value is substantially affected by sample size, and *p* values from small samples are extremely unreliable (Halsey et al., 2015). On the other hand, if the sample size is large enough, almost all effects will be deemed to be statistically significant, even if they are small, or practically or clinically meaningless. In essence, with large samples we may encounter a situation where all effects are significant, even if none are *significant*. We are in an era of big data that paradoxically accompanies a replication crisis (Open Science, 2015) in a number of scientific disciplines. Thus both issues, interpreting effects in the context of both small and large samples, are relevant for contemporary scientific investigations. Indeed, the global definitions of small and large are arbitrary. In reality, the meaningfulness of effects, as well as the sufficiency of sample size and its relation to effect size, are context dependent.

### 2.2 Separating the size of the effect from the error term

To shift our focus to the size of the effect, we need to take a look at a couple of simple mathematical equations. We will return to our heights example to anchor this discussion, using data on male and female heights from the CDC-NCHS nationally representative anthropometric survey for 19-year-olds.^1^ Our goal here in examining these equations is to separate the two important pieces of statistical information from each other, the size of the effect and the error term, and set up a discussion of the more important of the two, the size of the effect and how to interpret it. We will also examine how sample size affects specifically the calculation of the error term and the significance test.

**Fig. 1:**
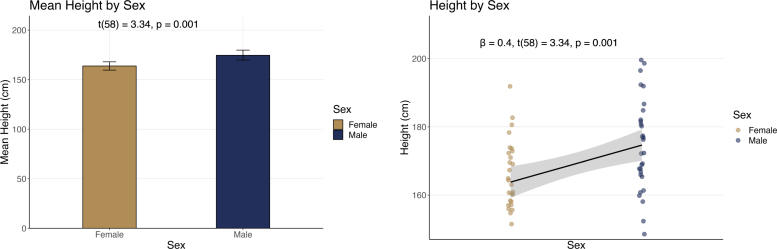
Two complementary ways of analyzing the same data will give identical results for statistical significance, but different standardized effect size estimates. On the left, the independent samples *t*-test of means will show a significant effect. The appropriate standardized effect size is typically Cohen’s *d* or complementary alternative. On the right, the *t*-test of the standardized slope difference against zero provides the same result, and the effect size estimate of β and $r_{\text{pb}}$. The latter approach has analogies in the general and generalized linear model frameworks. Although the metrics differ, they are simply different ways of summarizing the size of the effect, and thus provide the same information in different ways.

For our height example, to conduct the statistical test of the difference, most researchers learn to apply a *t*-test of independent sample mean differences. However, we could also approach this from a linear model framework in which the predictor in the equation is a categorical variable of male vs. female, or simply compute the correlation between the categorical predictor and the outcome (a special case of the Pearson correlation, the point-biserial correlation; $r_{\text{pb}}$). These are slightly different ways of examining the data and emphasize different aspects. For example, to plot the data in Fig. 1 we took a sample of *n* = 30 males and *n* = 30 females from the CDC-NCHS survey data. Here we can emphasize the difference between the means (on the left), or the association between the variables (on the right). But if we conduct a statistical test, we get the exact same answer for the significance test. Thus, in Fig. 1 we see the *t*-test of means (assuming equal variance) provides the same *t*-value and *p*-value as the test of the regression slope against zero. Furthermore, the correlation coefficient ($r_{\text{pb}}$ = 0.40) is the same as the standardized regression slope, β.^2^

The equation to assess statistical significance, in all three cases, is a ratio of the size of an effect (noted in blue in Eqs. (1)–(3)) relative to an estimate of the standard deviation of the sampling distribution of the effect (the standard error term, noted in red in Eqs. (1)–(3)). *Effect size* refers to the magnitude of the association between variables, or the degree to which the phenomenon under study is manifested (Cohen, 1988). One can also refer to standardized effect sizes (i.e., specific standardized metrics).

Consider the denominator (the standard error). In all three formulas below, the sample size *n* appears under a square root; therefore, as *n* increases the standard error decreases in proportion to $1/\sqrt{n}$. This dependence on *n* is separate from the data’s variability (the standard deviation terms) and from the effect size in the numerator (blue). Consequently, under the alternative hypothesis with a fixed nonzero population effect, shrinking standard error makes the expected magnitude of t grow roughly like $\sqrt{n}$, so *p*-values are smaller on average (i.e., power increases). This makes sample size a key component of statistical power, which in the NHST framework is defined as the probability of rejecting a false null hypothesis. With the Type I error rate held constant (at α = .05), the magnitude of the population parameter (e.g., the difference between means) depends on the actual effect being estimated. Enhanced power primarily relies on more precise parameter estimates facilitated by improved study measures and designs, and by increased sample size (Rothman et al., 2008, Button et al., 2013, Hong and Park, 2012).

(1)
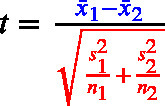

(2)
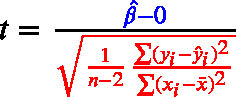

(3)
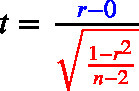


Rosenthal and DiMatteo (2001) summarize this succinctly as:

Significance Test=Effect Size×Study Size

Even a very small effect size can produce a statistically significant result if the study size is large enough. Conversely, a large effect size in a small sample may also reach significance, but such results are often less stable and more prone to overestimation because of sampling variability. This highlights the importance of considering both components, the magnitude of the effect and the sample size, when interpreting statistical significance.

### 2.3 Common effect size measures

Increasing sample size is obviously a good goal because as sample size increases there is a concomitant decrease in the standard error estimate. This means precision of the population estimate improves. However, at a certain point this leads to diminishing returns–the estimate is precise, and reductions in *p* are somewhat misleading as an indicator of precision. We are really interested in the population effect, from the top of the equation. Furthermore, we can begin to derive estimates of the magnitude of the difference by examining *effect size*. Effect size tells us something very different from the *p*-value: it can, interpreted in context, tell us about the practical and potentially clinical importance of the finding. In Cohen’s (1988) terms, the effect size is “the *degree* to which the phenomenon is present in the population” (p. 9), but there are also more nuanced definitions of the term (Kelley and Preacher, 2012a, Peng and Chen, 2014).

There exist numerous methods for providing standardized effect size estimates, each with its own advantages and preferred applications based on statistical considerations, such as base-rate difference effects and statistical robustness. However, digging into a comprehensive review of these various measures would risk overwhelming the reader (for an extensive treatment, see Kelley and Preacher, 2012a, Rosenthal et al., 2000, Babchishin and Helmus, 2016, Peng and Chen, 2014, McGrath and Meyer, 2006, Gignac and Szodorai, 2016, Valentine et al., 2019, Ferguson, 2009a, Grissom and Kim, 2005). Following the lead of Rosenthal et al. (2000), we will center our discussion on correlation coefficients, denoted as the “*r*-family” measures. These standardized metrics of association facilitate comparison across different studies and are likely familiar to most readers. Consequently, we will primarily focus on the *r* effect size, which applies to simple correlations as well as effects estimated within both the general and generalized linear model frameworks. Nonetheless, we will also explore several other specific effect size metrics that extend beyond the *r*-family measures. Thus, before diving into these, it is essential to revisit one of the most widely used effect size measures in the social sciences, Cohen’s *d* (Kelley and Preacher, 2012a, Cohen, 1988).

#### 2.3.1 Cohen’s *d*

Cohen’s *d* is the standardized mean difference estimate of the population parameter mean difference δ, which is taken as:

(4) $$d=\frac{\text{Mean difference}}{\text{Pooled standard deviation}}=\frac{\left|{\overset{¯}{x}}_{1}-{\overset{¯}{x}}_{2}\right|}{\sqrt{\frac{s_{1}^{2}+s_{2}^{2}}{2}}}$$

Related measures, like Hedge’s *g* (Hedges and Olkin, 1984), provide a modified calculation of the standard deviation estimate to correct for sampling bias. Furthermore, it is important to note that estimation of *d* is problematic when there is heterogeneity of variance across the groups (Grissom and Kim, 2001), and there are alternative calculations (see Peng and Chen, 2014). However, we will keep it simple in this review and focus on just a few effect sizes to drive home key concepts. It is also worth pausing to compare the calculation of *d* in Eq. (4) with the *t*-test of means in Eq. (1). The main difference is the inclusion of sample size in the denominator for the *t*-test of means. Cohen’s *d* remains unaffected by changes in sample size once the standard deviation values are determined.

Cohen (1988) provides guidelines for small (0.2), medium (0.5), and large (0.8) effects. It has been repeatedly emphasized that these are guidelines (Cohen, 1988)–they are provided independent of context. Whether such effects actually are small, medium, or large really depends on the research question and the context. In our heights example (with *n* = 30), the effect size is about *d* = 0.86, which is in the large range according to the guidelines.

**Fig. 2:**
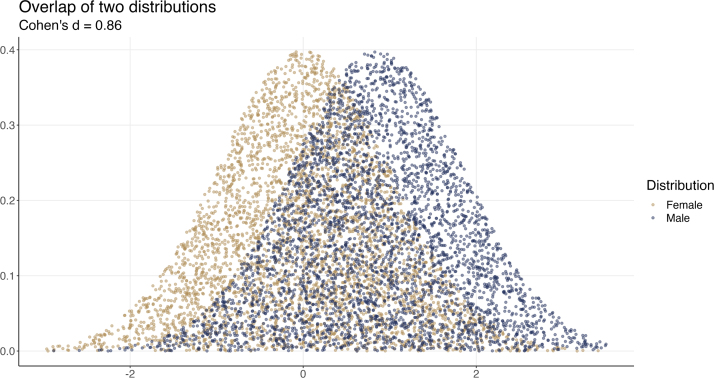
Simulated data of two distributions based on an effect size of Cohen’s *d*= 0.86. Each point represents one person.

How can we contextualize this effect size? Following the formulas spelled out in Cohen, 1988, Reiser and Faraggi, 1999, Ruscio and Mullen, 2012, Grissom and Kim, 2005 and Magnusson (2023) provide a nice organization for understanding these effects in what are called “common language terms”. Thus, in common language terms, we can say over 79.7% of males will be above females in terms of height (i.e., Cohen’s $U_{3}$ metric), and about 67.8% of the distributions will overlap. If a person is picked at random from these two samples, there is a 72.1% chance a male height will be higher (i.e., this is the “probability of superiority”) (Magnusson, 2023, Reiser and Faraggi, 1999, Ruscio, 2008, Grissom and Kim, 2001, Grissom and Kim, 2005). We can also see this visually with simulated data, based on the computed effect size, in Fig. 2. This fits generally with our common-sense understanding of heights in the human population–men tend to be taller than women, but there is a lot of overlap. At the same time, more men are on the “taller” end of the distribution, and more women are on the “shorter” end.

We can also look at whether our estimates from *n* = 30 are good. Our small sample example was drawn from the larger publicly available sample data. From our small sample, the mean estimates were off by about 1–2 cm from the larger sample data: 163.8 cm for females (compared to the estimate from the larger sample of 161.3), and 174.7 cm for males (compared to the estimate from the larger sample of 175.3). Furthermore, the effect size from the small sample is an underestimate of effect size from the larger sample, *d* = 1.03, which illustrates the error contributed by sampling variability.

#### 2.3.2 *R* effect size

An alternative measure of this effect is *r*, which we can calculate with a simple conversion, provided the sample sizes are equal (see McGrath and Meyer, 2006 for an in-depth discussion of this issue and base-rate dependence of the measures).

(5) $$r=\frac{d}{\sqrt{d^{2}+4}}$$

Not surprisingly, this conversion gives us the correlation coefficient we calculated earlier from the data (*r* = .40, which is also equal to the β for a predictor with two levels and equal sample sizes). In Cohen’s guidelines, *r* = 0.1 (small), 0.3 (medium), and 0.5 (large) are usually given (although some adjustment is given for the special case of the point-biserial *r*, see Section 3.2 of Cohen, 1988). *r* can also be squared to calculate a percent variance. This is the proportion of variance in either of the two variables that can be predicted (or accounted for) by the variance of the other variable. For example, an *r* = 0.5 computes to $r^{2}$ = 0.25, indicating that 25% of the variance in variable one can be attributed to the variance in variable two (although see Funder and Ozer, 2019).

#### 2.3.3 Semipartial correlation $r_{sp}$

An advantage of *r* is that it is applicable beyond the simple comparison of means or simple bivariate correlation situation. For example, each predictor in a multiple regression is tested to determine if the slope (*b*) is different from zero. A useful correlation effect size for these situations is the *semipartial correlation*, $r_{sp}$. In a multiple regression, the β is a standardized effect size derived from *z* scores of the outcome variable and the regressors, which makes the index independent of the units of measurement (i.e., the slope is the expected change in standardized units of the outcome predicted by standardized units of the predictors). The β is considered by some to be an effect size in its own right (Kelley and Preacher, 2012a), but often researchers are interested in answering the following question: “What is the association between my variable of interest and the outcome variable, after controlling for the other covariates in the model?” In a multiple regression framework, this is what the $r_{sp}$ provides (Abdi, 2007)–i.e., an estimate of the specific portion of variance explained by a given independent variable in a model that also contains other predictors. In addition to dissociating the partial correlation from the semipartial correlation, Kim (2015) gives a nice overview and examples for simple models, such as when a third variable (Z) is correlated with Y or both X and Y. But it gets more complicated for regression equations with multiple covariate terms in the model, which are often suggested for analysis of ABCD data (Dick et al., 2021), or when multilevel models are considered. The reader is referred to specific citations for approaches to the calculation of $r_{sp}$ for these models (Stoffel et al., 2021, Nakagawa et al., 2017, Rights and Sterba, 2020, Rights and Sterba, 2019). It is sufficient to state that, given careful modeling practice and attention to statistical challenges inherent in multiple regression analysis (Lynam et al., 2006), the $r_{sp}$ is a straightforward effect size measure for multiple regression frameworks. It explains the total variation of the outcome variable that is uniquely explained by the predictor of interest. Squaring the $r_{sp}$ also gives the percentage of unique variance explained by the predictor of interest on the outcome variable, beyond the proportion already explained by other variables in the model.

#### 2.3.4 Effect sizes for dichotomous outcomes: Odds and risk ratios

So far, our focus has been on continuous outcomes. However, there are also important effect size measures tailored for dichotomous outcomes. Two such measures, commonly employed in medical and clinical research but potentially applicable to the analysis of ABCD data, are odds and risk ratios. In medical and clinical contexts, these metrics typically assess associations within intervention scenarios, reflecting the risk or odds of a specific outcome. Statistically, a risk represents the probability of an outcome occurring among all possible outcomes, while odds, though related, denote the probability of an event occurring relative to the probability of it not occurring (Ranganathan et al., 2015). The risk ratio (RR) compares the risk of an event between two groups (e.g., exposed versus unexposed), whereas the odds ratio (OR) compares the odds of an event between the same groups. An RR (or OR) of 1.0 indicates no difference in risk (or odds) between the groups being compared. Values above 1.0 signify a higher risk (or odds) in the exposed group, while values below 1.0 indicate a lower risk (or odds) in the exposed group (Ranganathan et al., 2015).

(6) $$\text{OR}=\frac{\text{odds of exposure in the case group}}{\text{odds of exposure in the control group}}=\frac{a/b}{c/d}=\frac{ad}{bc}$$

where:

*a* is the number of exposed cases,

*b* is the number of unexposed cases,

*c* is the number of exposed controls,

and *d* is the number of unexposed controls.

(7) $$\text{RR}=\frac{\text{risk of outcome in the exposed group}}{\text{risk of outcome in the unexposed group}}=\frac{a/\left(a+b\right)}{c/\left(c+d\right)}$$

where:

*a* is the number of exposed individuals with the outcome,

*b* is the number of exposed individuals without the outcome,

*c* is the number of unexposed individuals with the outcome,

and *d* is the number of unexposed individuals without the outcome.

These measures are frequently employed in epidemiological and medical research, domains known for use of large sample sizes. Consequently, the question of what constitutes a meaningful interpretation of these measures has been a topic of discussion within these fields. For instance, Chen and colleagues (Chen et al., 2010) attempted to anchor the interpretation of ORs by relating them to Cohen’s *d* effect sizes. They proposed that ORs of 1.68, 3.47, and 6.71 correspond to Cohen’s *d* values of 0.2 (small), 0.5 (medium), and 0.8 (large). However, as previously noted, the meaningfulness of these interpretations is contingent upon the context in which they are applied. Others have highlighted the potential pitfalls of relying solely on ORs and RRs as measures of effect size, especially when discussing clinical significance. Without agreed-upon standards, and considering the influence of base-risk, these measures can sometimes lead to misleading conclusions (Kraemer et al., 2003, Ferguson, 2009a). For instance, a twofold increase in risk may carry different implications depending on the baseline risk level (e.g., 10% versus 1%). Nevertheless, these measures offer the advantage of being readily interpretable in layperson’s terms. For instance, an OR indicates “My odds are x times greater than”, while an RR suggests “An event is x times as likely”. This accessibility allows for straightforward communication of findings. For example, an RR of 1.2 implies that exposed individuals are 20% more likely to develop a disease, while an OR of 1.2 indicates a 20% increase in the odds of disease occurrence. To illustrate, the RR for lung cancer among male smokers is approximately 23, meaning that male smokers are 23 times more likely to develop lung cancer than male nonsmokers (Ferguson, 2009a). This practice of providing a “common language explanation” is recommended for reporting ABCD data.

With several common effect sizes now addressed (summarized in Table 1), it is beneficial to take a slight detour and mine further into the issue of sample size. Initially, we will explore the ramifications of small sample sizes on statistical power, highlighting why they pose unique challenges for statistical inference. However, it is important to note that large sample sizes also introduce their own set of complexities for inferential statistics, albeit with a shift in focus from inference issues to the challenges of interpreting whether observed effects merit attention. Consequently, in subsequent sections, we will examine examples from the ABCD study, offering recommendations that emphasize interpreting effect sizes within the context of “statistical overpowering” induced by large samples.

**Table 1:** Some common effect size and strength of association measures.

Effect size	Formula	Description
d	$\frac{\left|{\overset{¯}{x}}_{1}-{\overset{¯}{x}}_{2}\right|}{\sqrt{\frac{s_{1}^{2}+s_{2}^{2}}{2}}}$	Cohen’s *d* measures the standardized difference between two means.

r	$\frac{\sum \left(X_{i}-\overset{¯}{X}\right)\left(Y_{i}-\overset{¯}{Y}\right)}{\sqrt{\sum {\left(X_{i}-\overset{¯}{X}\right)}^{2}\sum {\left(Y_{i}-\overset{¯}{Y}\right)}^{2}}}$	Pearson *r* measures the strength and direction of a linear relationship between two variables.

$r_{pb}$	$\frac{{\overset{¯}{x}}_{1}-{\overset{¯}{x}}_{2}}{\sqrt{\frac{s_{1}^{2}\left(n_{1}-1\right)+s_{2}^{2}\left(n_{2}-1\right)}{n_{1}+n_{2}-2}}}\sqrt{\frac{n_{1}n_{2}}{n}}$	Point-biserial *r* measures the correlation between a continuous variable and a binary variable, where ${\overset{¯}{x}}_{1}$ and ${\overset{¯}{x}}_{2}$ are the means of the two groups, $s_{1}$ and $s_{2}$ are the standard deviations of the two groups, and $n_{1}$, $n_{2}$, and n are the sample sizes.

$r_{sp}$	$\frac{r_{YX.PQ}}{\sqrt{1-r_{YX.PQ}^{2}}}$	Semipartial *r* measures the unique contribution of a predictor to the explained variance in the outcome variable, while controlling for the effects of other predictors. Where $r_{YX.PQ}$ is the correlation between the outcome variable (Y) and the predictor of interest (X), controlling for the effects of other predictors (P and Q).

β	$\frac{\sum_{i=1}^{n}\left(x_{ij}-{\overset{¯}{x}}_{j}\right)\left(y_{i}-\overset{¯}{y}\right)}{\sum_{i=1}^{n}{\left(x_{ij}-{\overset{¯}{x}}_{j}\right)}^{2}}$	Beta is the standardized coefficient in a regression equation, representing the change in the dependent variable for a one-standard-deviation change in the predictor variable.

Odds ratio	$\frac{ad}{bc}$	Odds ratio measures the likelihood of an event occurring in one group compared to another group, where a is the number of exposed cases, b is the number of unexposed cases, c is the number of exposed controls, and d is the number of unexposed controls.

Risk ratio	$\frac{a/\left(a+b\right)}{c/\left(c+d\right)}$	Risk ratio measures the relative risk of an event occurring in one group compared to another group, where a is the number of exposed individuals with the outcome, b is the number of exposed individuals without the outcome, c is the number of unexposed individuals with the outcome, and d is the number of unexposed individuals without the outcome.

## 3 The problem with small samples

The conventional wisdom surrounding sample size suggests that researchers aim for a minimum of 30 participants (*n*; per group for categorical predictors). However, the origins of this recommendation are somewhat murky. Speculations range from the convenience of the *t*-distribution approximating the normal distribution when degrees of freedom (*df*) are around 30, to the practicality of *t*-tables fitting neatly onto textbook pages for *df* = 30 (for example, see Table IV of Fisher’s early 1925 classic *Statistical Methods for Research Workers* Fisher, 1925). Regardless of the origin, there is no magic in *n* = 30 or any other specific sample size. It is crucial to recognize that what truly matters is statistical power. Cohen, 1990, Cohen, 1988 illustrates that even with *n* = 30 per group and a significance level α = .05, the power to detect a medium-sized effect is merely 0.48—essentially a coin flip. Considering neuroimaging research, where effect sizes tend to be smaller, Button et al. (2013) found that studies with a sample size of around 30 (*n* = 28) had a median power of less than 31%.

**Fig. 3:**
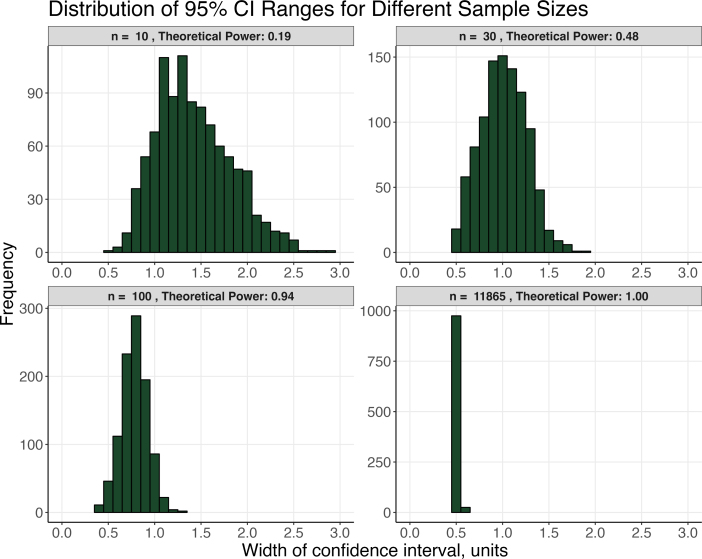
From Halsey et al. (2015), this simulation shows larger sample sizes estimate effect sizes more precisely. Random samples were drawn for each *n* based on an effect size of δ= .5 (1000 iterations). Histograms show the distributions of 95% CI ranges for different sample sizes. As sample size increases, the distribution more faithfully estimates the population effect size. The last figure for *n*= 11 865 shows how precisely the effect is estimated for the ABCD sample size.

In one sense, statistical power is a statement about replicability, and low-powered studies have poor replicability–indeed, *p* values are only as reliable as the sample from which they are collected (Halsey et al., 2015). In a simulation, Halsey and colleagues (Halsey et al., 2015) showed the wide distribution of confidence intervals for small samples. Fig. 3, which replicates their simulation, was generated by creating two hypothetical population distributions differing by an effect size of 0.5, each with a standard deviation of 1. By repeatedly sampling from these distributions and plotting the 95% confidence interval range, we gain a visual understanding of the extent of error in estimating the true difference (*d* = 0.5). For a large sample, such as the size of the ABCD study (*n* = 11,865), accurate estimation of the population difference is consistently achieved (power = 1.00), as evidenced by the histogram tightly clustered around 0.5. However, for small samples like *n* = 10 and *n* = 30, substantial variability exists, with estimates differing by 0.6–0.7 orders of magnitude. In some instances, confidence intervals may extend as high as 2.0–3.0 instead of remaining close to 0.5. Moreover, Halsey et al. (2015) demonstrated that there is notable variability in *p*-values calculated from underpowered samples. Their study revealed that only samples with a power exceeding 0.90 consistently returned similar *p*-values through repeated simulations; otherwise, the range of *p*-values derived from such samples was unacceptably wide.

Halsey and colleagues’ (Halsey et al., 2015) simulation highlights yet another unsettling reality concerning underpowered studies stemming from small sample sizes. In scenarios of low statistical power, *p*-values below the nominal α level are only observed when the sampled data are relatively extreme—a circumstance inherently unlikely to occur. When the field predominantly publishes results that achieve statistical significance at the nominal α level, it fosters an inflated perception of the true effect within the population. This phenomenon is a fusion of two distinct issues. The first is the “file drawer problem” (Rosenthal, 1979), which illustrates that studies yielding null results often go unpublished, residing perpetually in the proverbial “file drawer”. The second is the “winner’s curse” (Ioannidis, 2005), which labels the alternative—namely, that only statistically significant findings, or the “winners”, find their way into the literature. However, when these findings are derived from small samples, they tend to overestimate the population effect, sometimes to a significant degree.

To what extent does this pose a challenge in psychological and cognitive neuroscience research? The replication crisis in psychological science (Shrout and Rodgers, 2018) suggests it may be quite significant. Factors such as publication bias and researchers’ incentives to uncover significant associations (Button et al., 2013, Simonsohn et al., 2014), compounded by the prevalent use of small sample sizes in the literature, imply that reported population effect sizes may be considerably larger than reality (Paulus and Thompson, 2019, Kendler, 2019). Analyses of existing literature lend weight to this concern (Bakker et al., 2012), suggesting that numerous published neurodevelopmental associations could potentially exhibit significantly exaggerated effect sizes (Button et al., 2013, Ioannidis, 2008). Ioannidis (2005) has even argued that a majority of research findings in the literature are false, although the specifics of these concerns are subject to debate (Ashton, 2018). Nevertheless, it is plausible that the actual connections observed in nature might be relatively modest. For instance, Meyer and colleagues (Meyer et al., 2001) examined 125 meta-analyses in psychology and psychiatry, revealing that most relationships between variables fall within the range of *r* = 0.15 to 0.3, with many effects even smaller. Gignac and Szodorai (2016) reported a similar range in their meta-analysis. Furthermore, Miller et al. (2016) analyzed associations between multimodal imaging and health-related outcomes in UKBiobank data, finding that even the most notable associations explained only around 1% of the variance in outcomes. Finally, Owens and colleagues (Owens et al., 2021) scrutinized correlations across 161 instruments administered in the ABCD study at baseline (ages 9–11 years). While most correlations were small by conventional standards, the range was instructive. For instance, the highest correlation, between height and weight, was *r* = 0.60. However, other correlations were likely attenuated by the age range under investigation. For instance, the association between age and weight was only 0.24, whereas age and height had a correlation of 0.43. This underscores the importance of contextualizing effect sizes within the specific study and measurement tools. Moreover, it is probable that these associations evolve as children progress through adolescence.

These concerns about the reliability and generalizability of findings from studies with small sample sizes carry significant implications, particularly in the realm of public health. Take, for instance, the infamous Wakefield study (Godlee et al., 2011), which suggested a link between the measles, mumps, and rubella (MMR) vaccine and autism based on data from just 12 children. Despite being retracted due to fraud and ethical violations (Godlee et al., 2011), one might argue that its small sample size alone should have raised red flags from the outset. Contrast this with a study published in *The Lancet* the same year, which found no such effect in a prospective analysis involving over 3 million vaccinated children (Peltola et al., 1998). This finding has been corroborated by numerous large-sample studies and meta-analyses (Taylor et al., 2014, Madsen et al., 2002). One wonders why the study with the small sample garnered so much attention.

The Wakefield study serves as an extreme example, yet this problem extends throughout the field. Indeed, small-scale studies may initially yield findings and theories that appear credible, only to falter under scrutiny with larger samples. Consequently, misdirected efforts and resource allocation can ensue, regardless of whether the findings pertain to public health or policy. Addressing this issue necessitates well-designed, large-sample research studies. However, such studies come with their own set of challenges. First, large sample studies have a financial cost, which obviously varies by the study, but is often much larger than more focused investigations. This is a barrier for a lot of research groups. Second, data sets can quickly become unwieldy, requiring researchers to stay abreast of new software developments to manage them effectively. Third, large-sample studies may not always be optimally designed to address the research question at hand and may be repurposed to do so. Although still valuable, this practice leaves room for more focused small-sample studies, provided they are sufficiently powered to yield reliable conclusions. Finally, and most relevant to the present discussion, researchers must shift their focus to prioritize effect size reporting and interpretation.

## 4 The “problem” with large samples

**Fig. 4:**
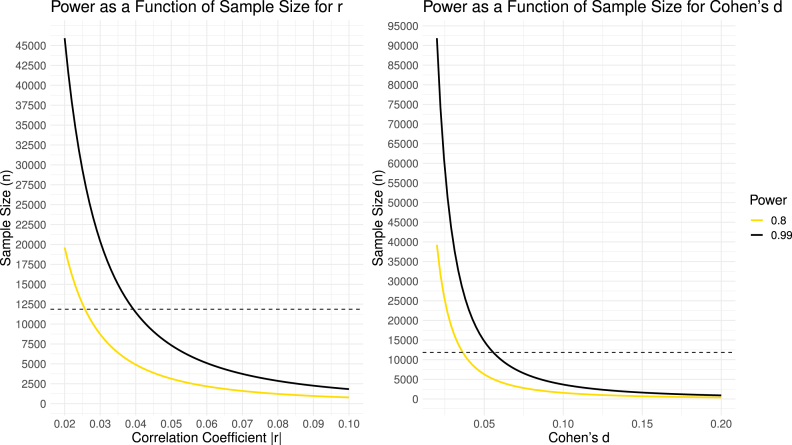
Power curves for *r* and *d* values for two different power levels, the standard 0.80, and 0.99. The dashed line is placed at the ABCD sample size on the y-axis. The far right of the x-axis is the *r* or *d* value considered by Cohen to be “small”.

If small samples pose challenges, large samples also present their own set of obstacles. Among these is the phenomenon often referred to as “statistical overpowering”. This occurs when the statistical power becomes so robust that even trivial effects register as statistically significant due to the high precision in estimating the population effect. Fig. 4 illustrates this through power curves depicting *r* and *d* values at two distinct power levels: the conventional 0.80 and the more stringent ’nearly assured power’ of 0.99. The dashed line marks the ABCD sample size on the y-axis. On the far right of the x-axis lies the threshold for what Cohen defines as a “small” effect. Remarkably, even for minute effects (e.g., *r* ≈.04), the ABCD sample size virtually guarantees statistical significance. As the effects amplify, significance becomes assured at increasingly smaller sample sizes. However, this is not a drawback (in fact, it is common in epidemiologic and genetic studies). Rather, it signifies that the sample’s effect size likely offers a precise estimate of the actual population effect, which, ultimately, is our primary concern.

In practical terms, this implies that in many analyses of ABCD data, emphasizing statistical significance becomes less relevant or revealing, while highlighting effect sizes and their interpretation becomes crucial. When statistical significance is easily attained with large samples, we encounter another dilemma. There comes a juncture where effect sizes are so minuscule that, even if they achieve statistical significance, they might not merit attention. However, pinpointing this threshold is context-dependent. It is valuable at this point to revisit some classic examples of small effects that carry meaning, and consider why they hold meaning.

### 4.1 What defines a small effect and when is an effect size “too small to be meaningful”?

Small effects, interpreted in the correct context, can be important when they impact large populations, relate to potentially important events (e.g., when the outcome is catastrophic, such as death), and/or if they systematically accrue over time (Funder and Ozer, 2019). One classic example of a very small but meaningful effect is given by Abelson (1985) and Funder and Ozer (2019). Abelson found that the association between the outcome of a single at-bat in major league baseball and the season batting average was tiny (*r* = 0.056)—a single batting performance explains 0.00317% of the variance of the season average. This seems counter-intuitive since good hitters should consistently hit well, and one would expect the association to be higher. However, although the association is quantitatively small, these small effects accrue over the course of the season, which makes them potentially important. Other examples of these potentially accruing small effects are provided in reviews (Funder and Ozer, 2019, Gotz et al., 2022, Primbs et al., 2023, Meyer et al., 2001), and include the association between extroverted personality traits and shopping expenditure (*r* = 0.09; Weston et al., 2019), calcium intake and bone mass (*r* = 0.08; Welten et al., 1995), and the associations between drugs like aspirin and propranolol and heart attack risk (ϕ*’s* = 0.03 and 0.04; Rosenthal, 1990). The latter two are good examples because they are justified often by low cost and small risk of side effects, so even a small effect is worthwhile (e.g., taking aspirin after a heart attack prevents 85 heart attacks for every 10,845 people Rosenthal, 1990).

Heart attack risk examples also illustrate how effect sizes can be interpreted. For example, Ferguson (2009b) notes that when events are rare or base rates are low, it is more appropriate to calculate effect sizes *only for the subset of treated individuals* with the condition. Using the Salk vaccine as an example, Rosnow and Rosenthal (2003) calculated an overall effect size of *r* = .011 (with $r^{2}$ = .0001) across vaccinated and unvaccinated individuals—effectively zero. This would seem to indicate little benefit to the vaccine. But Ferguson argues that this is because the incidence of polio is extremely low in the general population, diluting the effect. Instead, he argued that the effect should be evaluated among hypothesis-relevant cases—those who are actually exposed to the virus. Within this group, the effect size for vaccinated individuals rises dramatically to *r* = 0.74. This illustrates how calculating hypothesis-relevant effect sizes is fundamentally a matter of interpretation within the study context.

Still, given that virtually all effects in the social and medical sciences are non-zero – at least in correlational studies and outside highly controlled experiments (Kirk, 1996, Tukey, 1991) – an important question arises: at what point is an effect “too small to be meaningful”? This issue remains the subject of active debate (Matz et al., 2017). For example, Götz and colleagues advocate for moving away from an expectation of large effects in the social sciences, urging a focus on reporting accurate and *meaningful* effects (Gotz et al., 2022). In response, Primbs and colleagues ask: what constitutes “meaningful”? They argue that attributing meaning to small effects requires robust evidence and a clear, falsifiable rationale (Primbs et al., 2023). Moreover, it is common for researchers to lean on the notion of cumulative effects when confronted with small effect sizes. While such accumulation can be real (as in the Abelson example, or selection coefficients describing small adaptive changes over evolutionary time, Bersaglieri et al., 2004), its generalizability to other small effects requires additional empirical support. As Meehl observed, when nearly everything in the social sciences is correlated with everything else (Meehl, 1990), establishing criteria for meaningfulness becomes particularly crucial.

Ferguson (2009b) further suggests that outcomes with highly valid or perfectly measured endpoints (e.g., death or disease status) offer a benchmark for evaluating effect sizes in domains like social science, where measurement error is higher and construct validity is more variable. In this context, Ferguson (2009a) proposes thresholds for practically significant effects: a “recommended minimum” effect size of *r* = 0.20 or *d* = 0.41, with moderate and large effects defined as *r* = 0.5 and 0.8 (*d* = 1.15 and 2.70), respectively. While these are useful benchmarks, even Ferguson (2009a) admits that “for effects with highly valid dependent measures (e.g., death) and using rigorous controlled outcome trials, lower values may have practical value” (p. 533).

An alternative approach is to use empirically derived guidelines specific to a subdiscipline. For instance, Lovakov and Agadullina (2021) analyzed effect size distributions in social psychology and found that the 25th, 50th, and 75th percentiles corresponded to *r* values of 0.12, 0.24, and 0.41 and Cohen’s *d* values of 0.15, 0.36, and 0.65, respectively. Brydges (2019) performed a similar analysis in gerontology, finding that the corresponding *r* values were 0.12, 0.20, and 0.32, and Hedges’ *g* values were 0.16, 0.38, and 0.76. Owens and colleagues (Owens et al., 2021), using the ABCD dataset, found effect sizes tended to be small by conventional standards (median *r* = .05; first and third quartiles = .03 and .09).

A valuable approach to evaluating whether an effect is “too small to be meaningful” is to directly define a SESOI (Lakens, 2022). A SESOI can be grounded in theory, prior research, cost–benefit considerations, outcome validity, or discipline-specific norms. For example, benchmarks derived from effect size distributions in the literature, computational models, or pilot studies can serve as guides (Primbs et al., 2023, Lakens et al., 2018, Mesquida and Lakens, 2024). As Mesquida and Lakens (2024) note, “unlike absolute thresholds, determining a SESOI allows for the meaningful interpretation of effect size while considering the contextual factors of the research...stating that an effect size is smaller than theoretically predicted, larger than an effect observed in a study that differs on one crucial factor, or large enough to adopt an intervention in practice improves statistical inferences” (p. 241). Once justified, the SESOI can be pre-registered or declared in advance of analysis. Importantly, it can be tested statistically using equivalence testing (Lakens et al., 2018, Lakens, 2017), which determines whether the observed effect falls within or exceeds the specified bounds. We illustrate this approach using ABCD data in Example 1.

A complementary approach that emphasizes effect sizes is the use of “estimation graphics” (Ho et al., 2019). These visualizations shift attention from the question of whether an effect exists to how large it is. Gardner and Altman (1986) first introduced the format: raw data are plotted on the left, while the effect size and its confidence interval appear on the right. Calin-Jageman and Cumming, 2019a, Cumming and Calin-Jageman, 2024, Calin-Jageman and Cumming, 2019b, and Ho et al. (2019), have since expanded this approach to visualize estimate uncertainty using permutation and bootstrapping. We illustrate how estimation graphics may be applied to ABCD data in Example 2.

Finally, the notion of “clinical significance” remains vital, especially in clinical, medical, and intervention sciences, where decisions often hinge on a cost–benefit analysis—namely, the degree of change required to justify the cost of a treatment (Keefe et al., 2013). We explore this concept further in the next section.

### 4.2 Defining “clinical significance”

In intervention science, a robust body of literature examines the intricacies of defining ’clinical significance’ or “clinically meaningful effects” (Keefe et al., 2013) in treatment studies. Kendall, for instance, defines clinical significance as the persuasiveness or meaningfulness of the magnitude of change (Kendall, 1999). Within treatment contexts, this notion translates to observable alterations in metrics related to the initial presenting issue, such as symptoms of inattention, resulting in participants returning to “normal” levels on those metrics. Often, “returning to normal levels” entails no longer meeting the clinical cutoff on a standardized instrument. To assess this, researchers commonly construct contingency tables based on participants transitioning between cells from pre- to post-treatment or on- and off-medication statuses. Traditionally, such assessments rely on statistical tests like the McNemar test for 2 × 2 contingency tables. However, emerging methodologies offer more comprehensive approaches to evaluate clinically significant change, such as *normative sample comparison* and the *reliable change index*.

Normative comparisons, as described by Kendall et al. (1999), involve several steps. Initially, researchers select a normative (unaffected) group for comparison post-treatment. Typically, established measures like the Child Behavior Checklist, provide normative data for this purpose, although it may be necessary to gather such data independently. However, a statistical challenge arises as researchers must, in a sense, establish the null hypothesis, indicating no difference at post-treatment between the treatment and normative groups. One approach to tackle this challenge is through the equivalency testing framework. Here, the null hypothesis posits that the difference between parameter estimates lies outside a predetermined interval of equivalence (δ). Hypothesis testing then assesses whether the difference between the treatment group at post-treatment and the normative group is significantly less than the predetermined threshold at which group differences would be interpreted as meaningful. Essentially, this framework enables the formulation of claims about group equivalency backed by statistical evidence. The normative comparison framework highlights how a treated group could exhibit statistically significant change (i.e., change from pre- to post-treatment scores reach *p* <.05 threshold) and yet not achieve clinical significance (i.e., post-treatment scores are not returned to “normative” levels).

While the normative sample comparison method enables us to explore whether treated individuals deviate at post-treatment from the normative sample concerning the measured symptoms, the reliable change index (Jacobson et al., 1984, Jacobson and Truax, 1991) tackles the question of whether the extent of change observed is substantial enough to be deemed meaningful. This approach involves assessing the number of participants transitioning from a dysfunctional to a normative range. The process entails categorizing difference scores based on the probability that a difference equal to or greater than the observed one could occur by chance alone. To accomplish this, researchers rely on an estimation of measurement error obtained from external data. Specifically, they calculate a difference score (post-treatment minus pre-treatment) divided by the standard error of measurement (Eq. (6)).

(8) $$RCI=\frac{x_{2}-x_{1}}{\sqrt{2{\left(s_{1}\sqrt{1-r_{xx}}\right)}^{2}}}$$

Here, $x_{1}$ and $x_{2}$ represent the observed scores at Times 1 and 2, respectively. The reliability coefficient $r_{xx}$ is typically derived from separate psychometric analyses of the measurement instrument, indicating the consistency or stability of scores obtained from the instrument. Additionally, $s_{1}$ denotes the standard deviation calculated from the data or, in cases with limited data points, reported from previous studies employing the same measurement instrument. Similar to our earlier equations, this ratio presents a comparison of the difference (effect) against an error term. In contrast to the normative comparison approach to assessing clinical significance, the RCI approach does not rely on an external “normative” (i.e., unaffected) sample. Rather the RCI approach uses the treatment group’s own pre-treatment scores to establish an amount of change from pre- to post-treatment that is large enough to be characterized as meaningful (in addition to simply statistically significant).

The reliability change index measures have sparked considerable debate, as evidenced by a recent comprehensive review (see McAleavey, 2024 for an in-depth exploration, including an extensive discussion of alternative measures). Originally designed for pre-post treatment designs and tailored to individuals rather than groups, the RCI is constrained by its reliance on only two measurements. Extensions allowing for more than two time points (McAleavey, 2022) might offer greater relevance for studies like ABCD. However, even in such cases, ABCD does not employ a treatment within an experimental framework. Consequently, the applicability of RCI and related metrics may be limited for the quasi-experimental nature of ABCD.

Of the two discussed metrics, then, only the normative sample comparison is likely to be relevant for ABCD. In this context, it is most likely to be applied to understand how symptoms might remit over the several years of the study relative to a normative comparison group, which indeed may be derived from the ABCD sample itself. In this way, analyses are akin to traditional equivalency testing frameworks (Ialongo, 2017; see Dick et al., 2019 and section 5.1 below for application to ABCD data). However, the concept of returning to group equivalency, which encompasses the notion of “clinically meaningful” outcomes, can seamlessly extend to practical implications examined in ABCD. In this sense, “practical significance” applies when we use these same methods to describe/assess findings related to developmental or academic variables outside of clinical or treatment settings.

## 5 Examples from the ABCD study

Up to this point, we have covered several key areas: (1) defining “meaningful effects” within the framework of the Frequentist Null Hypothesis Significance Testing (NHST) tradition, including common effect size definitions; (2) addressing the challenges posed by small sample sizes; (3) exploring the opportunities and complexities of large-sample studies; and (4) surveying definitions of clinical significance. These discussions lay the groundwork for a more principled interpretation of effect sizes in developmental science. We now turn to concrete examples drawn from the ABCD Study to demonstrate how these statistical concepts play out in practice. Each example serves as a case study in how to apply our proposed framework: to **define** a SESOI, **compare** observed effects to meaningful benchmarks, **test** whether effects exceed those thresholds using appropriate inferential tools, and **visualize** results to enhance interpretation and communication. These examples illustrate how a shift in focus – from statistical significance to practical relevance – can enrich our understanding of developmental phenomena and guide future research priorities.

### 5.1 Example 1: Bilingual advantages of executive function

In a previous study we explored predictions based on the “bilingual advantage theory” using data from the ABCD data release 1.0. This release had a sample size of 4524 participants (*n* = 2784 monolingual youth and 1740 self-reported bilingual youth) (Dick et al., 2019). This theory posits that, beyond the evident social and linguistic benefits of bilingualism, being bilingual might confer advantages in cognitive domains like executive function (Bialystok, 2001, Barac et al., 2014). While several meta-analyses suggest that such effects are minimal to non-existent in the general population (de Bruin et al., 2015, Lehtonen et al., 2018, Donnelly et al., 2019, Lowe et al., 2021, Gunnerud et al., 2020), there are also meta-analyses proposing bilingual effects (Grundy, 2020, Ware et al., 2020). The inherent vagueness of the theory further complicates matters, as it remains unclear which specific executive functions should exhibit benefits, compounded by the challenge of precisely defining bilingualism (Wagner et al., 2023). However, our focus here is not to untangle the nuances of the theory. Instead, we aim to narrow our example to illustrate how large sample sizes influence the interpretation of results characterized by small effect sizes, which nonetheless might tend to be statistically significant due to the large size of the sample.

In this example, we revisit analyses conducted in the original paper, but apply them to a larger sample drawn from the ABCD data release 5.1, specifically focusing on the baseline timepoint. Our expanded dataset now comprises 7351 monolingual youth and 4435 self-reported bilingual youth. Notably, our findings largely recapitulate the patterns observed in our previous report.

We examined four key outcome measures: NIH Toolbox Picture Vocabulary (“Vocabulary”), assessed in English, along with three executive function measures—Stop-Signal Reaction Time (SSRT; reverse-scored), NIH Toolbox Flanker (“Flanker”), and NIH Toolbox Card Sort (“Card Sort”). Higher scores indicate better performance across all outcomes. Our new analysis of bilingualism considered three distinct predictors based on self-reported language proficiency. Bilingual Status was dummy-coded as a binary variable, indicating whether individuals spoke another language (based on self-report). Bilingual Degree was also binary, but additionally reflected proficiency in speaking another language by indexing the frequency of self-reported usage of that other language with friends or family. Bilingual Use, an ordinal variable, was assessed solely among participants who identified as bilingual and ranged from 0 (minimal usage) to 8 (extensive usage) based on reported use of the other language with friends and family. For instance, the most proficient bilinguals self-reported speaking the other language with friends and family all the time.

The hypotheses outlined in Dick et al. (2019) suggest that bilingualism will be associated with improved performance on executive function measures. However, due to the necessity of learning the vocabularies of two languages, bilingual individuals are expected to exhibit worse performance on the English vocabulary measure, even though all ABCD youth are English speaking.

Table 2 presents the results of linear mixed-effects models, using complete cases for all variables. The first data column displays the effect size $r_{sp}$. Notably, all effects fall within the small to very small range according to Cohen’s standards (Cohen, 1988), ranging from —0.000—, explaining almost no variance, to —0.104—, explaining approximately 1% of the variance. Only the Vocabulary variables showed statistically significant associations. Indeed, all of the executive function measures hover around zero, and none reached statistical significance, even with the large ABCD sample size.

**Table 2:** Summary of models with $r_{sp}$, $R_{sp}^{2}$ with 95% CIs, and *p*.

Model	$r_{sp}$	$R_{sp}^{2}$	$R_{sp}^{2}$ 95% CI Lower	$R_{sp}^{2}$ 95% CI Upper	Degrees of freedom	*p*
Bilingual status: Vocabulary	0.000	0.000	0.000	0.000	10 270.1	0.99
Bilingual status: SSRT	−0.013	0.000	0.000	0.001	8804.9	0.21
Bilingual status: Flanker	0.018	0.000	0.000	0.001	9871.8	0.06
Bilingual status: Cardsort	0.001	0.000	0.000	0.000	8451.5	0.92
Bilingual degree: Vocabulary	−0.060	0.004	0.002	0.006	10 057.4	<0.0001⁎⁎⁎
Bilingual degree: SSRT	−0.009	0.000	0.000	0.001	8748.2	0.40
Bilingual degree: Flanker	−0.003	0.000	0.000	0.001	9496.1	0.80
Bilingual degree: Cardsort	−0.004	0.000	0.000	0.001	7154.8	0.67
Bilingual use: Vocabulary	−0.104	0.011	0.005	0.018	3409.3	<0.0001⁎⁎⁎
Bilingual use: SSRT	−0.017	0.000	0.000	0.003	3230.4	0.33
Bilingual use: Flanker	−0.026	0.001	0.000	0.003	3192.5	0.12
Bilingual use: Cardsort	−0.010	0.000	0.000	0.002	3696.6	0.54

Note. Effects are reported from linear mixed-effects models controlling for age, sex, genetic ancestry, education, income, reading, and fluid intelligence, with site nested within family as random effects. Bilingual status was dummy-coded as a binary variable indicating whether individuals spoke another language. Bilingual degree was also binary, reflecting proficiency in speaking another language and frequent usage with friends or family. Bilingual use, a continuous variable, was assessed only among participants who identified as bilingual and ranged from 0 (indicating minimal usage) to 8 (reflecting extensive usage) based on reported interactions with friends and family. SSRT was reverse scored so that for all outcomes, higher scores are better. Degrees of freedom and *p*-values are calculated according to the Kenward–Roger method (Luke, 2017, Kenward and Roger, 1997) and reflect complete-cases analysis.

*** *p*< .0001.

When accounting for the frequency of non-English language usage, a statistically significant – albeit small – association between bilingualism and diminished English vocabulary emerges, a finding both consistent with prior research (Hoff et al., 2012, Hoff and Core, 2015) and also face-valid (a person learning two languages might not incur a penalty in overall vocabulary across both languages (Hoff et al., 2012), but might divide that skill across the two languages, incurring a penalty for English vocabulary).

In contrast, none of the bilingual metrics yielded statistically significant associations with executive function measures. The minimal, near-zero effects suggest that these measured associations actually oscillate around zero, and except for Vocabulary, bilingualism does not have a notable effect on performance on these tasks.

How can we contextualize these effect sizes—even the non-significant ones? One approach, which we used in our original study (Dick et al., 2019), is to define a SESOI and evaluate observed effects against this threshold using an equivalence testing framework. This allows us to ask whether an observed effect is large enough to matter—not merely whether it differs from zero. Crucially, the SESOI should be defined *a priori*, based on theoretical, practical, or empirical justification. In the present context of bilingualism and vocabulary (and by extension, executive function), a reasonable SESOI might be the smallest effect that would reflect a meaningful disadvantage for a bilingual adolescent in academic or communicative settings. This could be anchored to known associations between standardized vocabulary scores and educational outcomes such as reading comprehension, academic risk, or eligibility for services. Prior studies document average vocabulary gaps between English learners and monolingual peers—typically ranging from 0.1 to 0.3 SD units, or roughly 1–5 points on the NIH Toolbox Vocabulary T-score scale (August et al., 2009, Hoff, 2013, Kieffer, 2008)–which have downstream effects on educational outcomes. In our original study (Dick et al., 2019), we operationalized the SESOI as β=±0.10, equivalent to a 1-point difference on the NIH Toolbox scale—what we consider the smallest potentially meaningful difference in this context. Given that two of the three executive function measures are also drawn from the NIH Toolbox, applying the same SESOI is a reasonable starting point, though domain-specific thresholds could also be justified.

Once the SESOI is defined, we can test whether an observed effect is small enough to be considered practically negligible—that is, effectively zero. This is done using an equivalence test (Lakens et al., 2018, Lakens, 2017), which reverses the typical logic of null hypothesis testing. Instead of testing whether the effect is different from zero, the null hypothesis posits that the effect lies *outside* the predefined equivalence bounds (δ). If the observed confidence interval falls entirely within the SESOI interval, we reject this null hypothesis and conclude that the effect is too small to be meaningful. In this way, equivalence testing allows us to statistically reject the presence of effects large enough to matter, shifting the focus from mere statistical detection to practical significance. This can be tested using a variety of methods, but one that has been proposed for regression models is the method by Anderson and Hauck (1983), validated using Monte Carlo estimation by Counsell and Cribbie (2015).

The proposed statistic is given by

(9) $$p=\phi \left[\frac{\left|b_{1}-b_{2}\right|-\delta }{s_{b_{1}-b_{2}}}\right]-\phi \left[\frac{-\left|b_{1}-b_{2}\right|-\delta }{s_{b_{1}-b_{2}}}\right],$$

where ϕ represents the standard normal probability function, δ represents the interval of equivalence (e.g., 0.1), b represents the regression coefficient, and s represents the standard error. When p ≤α, the null hypothesis of a difference is rejected. If the parameter estimate of the difference, along with its confidence interval, falls within the interval of equivalence, the parameter estimates are determined to be equivalent.

**Fig. 5:**
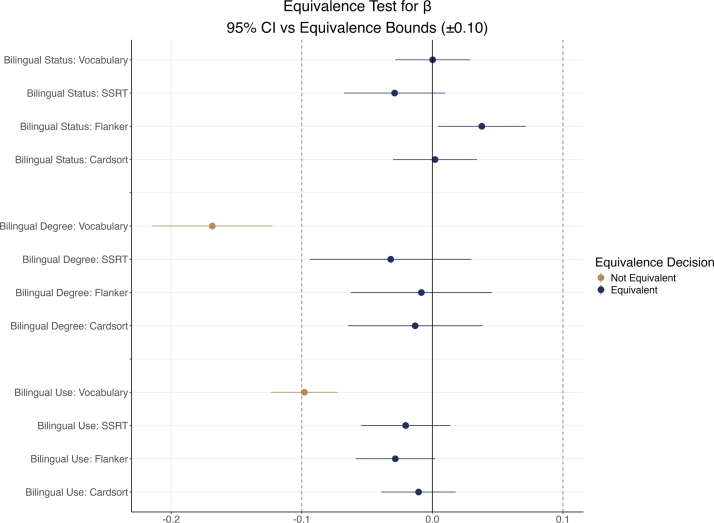
Results of the tests of equivalence for the standardized regression slope β. The figure shows the effect sizes (β) and CIs plotted against the interval of equivalence. Data points represent the parameter estimates (β) from the analysis for Table 2, along with the calculated CIs for the slope. To evidence statistical equivalence, CIs should be contained within the *a priori* defined interval of equivalence ($I_{\beta }^{-}$ to $I_{\beta }^{+}$), which was set to (−0.1 to 0.1). A bilingual advantage would show to the right of 0 on the x-axis, and any disadvantage to the left.

Fig. 5 shows the results. Only Bilingual Degree met the criterion for a practically meaningful effect: its 90% CI lay entirely beyond −.10. For Bilingual Use, the 90% CI touched the lower SESOI bound (near −.10), so the effect was neither equivalent (negligible) nor demonstrably meaningful; accordingly, we cannot reject effects that would still be considered meaningful. In this example the conclusions happen to align with the pattern of *p* values in Table 2, though these tests address different questions and need not coincide. This example also illustrates the value of visualizing the results. For example, one finding (from Bilingual Status predicting Flanker) was close to a significant effect in the initial analysis (*p* < .06), but sits comfortably within the defined SESOI bound, reinforcing the absence of a meaningful difference.

We can conclude this example by considering what it reveals about the interpretation of small effects in large-sample studies. As recent meta-analytical work underscores, even reliably observed effects may be practically trivial. For instance, Grundy (2020) asserts that “the overall effect size for the effects of bilingualism on EF performance is likely small, but positive and real” (p. 189). Yet notably, no effect size estimate or contextual benchmark is provided. As discussed earlier, nearly all effect sizes in large datasets will differ from zero to some degree—particularly when measurement error is minimized. The more meaningful question, then, is whether the observed effects are large enough to warrant further investigation or inform applied decisions. Claims of practical significance must be supported not only by statistical results, but by reference to empirical benchmarks and effect sizes with accompanying confidence intervals. Without such context, even well-meaning decisions – such as classroom placements or recommendations about home language use – could be influenced by statistically significant findings that, in truth, reflect negligible effects.

To illustrate this point more concretely, consider the β coefficient reported for the association between bilingualism and Card Sort performance in another large-sample study of children aged 5–7 yr (*n* = 11,250) (Hartanto et al., 2018). In that analysis, bilingualism predicted Card Sort scores with a β = 0.040. By comparison, socioeconomic status had a larger effect (β = 0.115), as did age (β = 0.075), English proficiency (β = 0.25), and even sex (β = 0.045). All predictors were statistically significant due to the large sample size, except for a variable “Asian Culture” (β = 0.010). This underscores the need to interpret effect sizes in context: statistical significance alone is insufficient. When examining ABCD or other large-scale data, we recommend situating observed effects alongside those from meta-analyses, similar large samples, and other variables within the same model. Visualization methods, such as plotting parameter estimates against SESOI bounds, can further support clear and principled interpretations of what constitutes a meaningful effect.

### 5.2 Example 2: Effects of prenatal cannabis exposure on fronto-limbic white matter

In our second example, we examine a recently published study by Evanski et al. (2024). Our aim here is not to review or critique the analytic methods or measures (see Baranger, 2024 instead), or to cast aspersions on the authors or the study. Rather, we view this as another instructive case highlighting the challenges we navigate as scientists in an era of big data.

Using the ABCD data, this study explored the effects of prenatal cannabis exposure (PCE) on brain development, specifically focusing on frontolimbic white matter pathways. In their analysis, they discovered that PCE was “associated with lower fractional anisotropy of the right (β = −0.005, *p* < .001) and left (β = −0.003, *p* = .007) fornix”. The authors duly note that they “demonstrated small, yet reliable, effects of PCE on white matter integrity during childhood, particularly in the fornix”. Importantly, the authors engaged in a responsible, purposeful, and thoughtful discussion of these findings, including an appropriate examination of effect sizes, and readers are encouraged to carefully consider the nuanced context presented in the Discussion section of their paper. For instance, they draw parallels between PCE and effects reported in the literature on prenatal alcohol exposure, and they link their findings to those reported by Owens and colleagues (Owens et al., 2021), who surveyed a range of associations in ABCD. The authors argue that their effects approximate the median effect size of that study. However, in our view, this is a generous interpretation. In fact, Table 2 from Owens et al. suggests that *r* values ranging from 0.01 to 0.04 indicate “below average” effects in ABCD. Regardless, Evanski et al. emphasize that these are “small yet reliable” effects. But the question arises: are they meaningful in the sense that we should be concerned about white matter development following PCE? And how can we contextualize these effects?

The SESOI and equivalence testing approach could (and arguably should) also be applied here. In addition, we argue that visual presentation of the effects is always helpful, so in this example, we provide two visualization methods that might compliment the statistical analysis. Let us begin with a visual depiction of the expected distribution of both groups based on the reported effect sizes. Although Cohen’s *d* was not reported in the paper, we can estimate it by considering the largest effect (adjusting for covariates, the effect of PCE on the right fornix was reported to be *r* = −0.04), giving us an approximate Cohen’s *d* = 0.08. With this estimation, we can calculate properties of the distributions and provide a visual overlap of these distributions through data simulation. It is anticipated that with this effect size, in common language terms 96.6% of the two groups will overlap, with 53.2% of the Unexposed group positioned above the PCE group. Furthermore, there is a 52.3% chance that a person picked at random from the Unexposed group will have a higher score than a person picked at random from the PCE group, which is basically a coin flip.

**Fig. 6:**
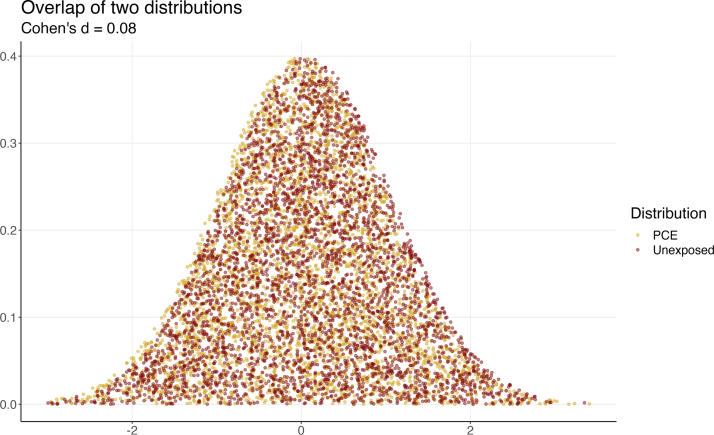
Simulated data of two distributions based on an effect size of Cohen’s *d*= 0.08. Each point represents one person.

Visually, Fig. 6 (based on simulations; Magnusson, 2023) starkly illustrates the overlap of the two distributions, which are largely indistinguishable. While this is simulated data derived from the reported effect size, it aligns with our expectations from examining Fig. 2 in Evanski et al. which demonstrates an average fractional anisotropy difference of .005 (note, fractional anisotropy (FA) metrics range from 0.0 to 1.0). We can further compare Fig. 6 of the present paper with Fig. 2 of the present paper, which illustrates a traditionally “large” effect. Within this broad context, one might argue that PCE is too small to meaningfully impact white matter diffusion properties in the reported pathways, despite the statistical significance of the effect.

**Fig. 7:**
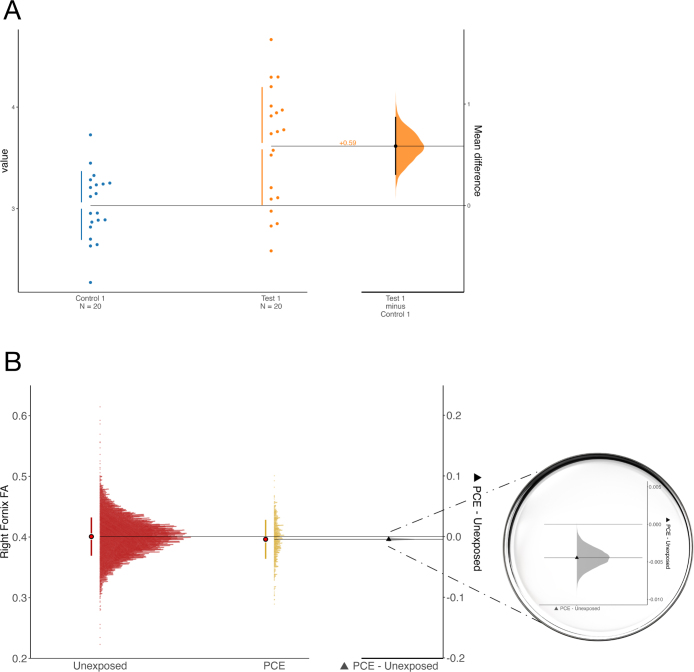
Estimation plots for simulated data (A), and for the data from Evanski et al. (2024) (B). In (A), the simulated data available from the dabestr() package are plotted, using the defaults provided in the help file. Bee swarm plots of this small toy sample are shown on the left, and on the right of this plot (in orange) is the bootstrap distribution of the mean difference effect size. In (B), we analyzed the data from Evanski et al. (2024). The left side of the plot shows the beeswarm plot of the data, emphasizing the distribution of data and the large size of the sample. On the right, the magnifying glass zooms in on the bootstrapped distribution of the effect size (mean difference; Δ). The default code was modified to add dots indicating the mean, and delta indicating the mean difference. (For interpretation of the references to color in this figure legend, the reader is referred to the web version of this article.)

To complement this interpretation, Fig. 7 presents an estimation plot comparing the effect of PCE on white matter integrity with simulated and real data. Panel A shows a toy example provided along with the dabestr package, which can be used to easily make estimation plots. The figure highlights visually a difference and distribution that separate comfortably from zero. Panel B applies this same approach to the Evanski et al. data, visualizing the effect of PCE on right fornix FA. The large beeswarm plot on the left emphasizes the breadth and variability of the data, while the zoomed-in delta plot on the right provides a magnified view of the small mean difference and its bootstrapped uncertainty. This dual representation allows readers to simultaneously grasp the reliability of the finding and its practical implications, reinforcing our broader theme: that visual tools, when paired with effect size estimation and inferential clarity, can enhance interpretability in large-sample studies. Such visualizations could be applied along with statistical analyses of the SESOI to highlight meaningful or lack of meaningful differences in an analyzed data set. While visualizations such as estimation plots can clarify the precision and practical size of effects, another powerful strategy is to compare effect sizes against external benchmarks—an approach exemplified in our third case study on structural brain differences in ADHD.

### 5.3 Example 3: Structural brain differences in Attention Deficit- Hyperactivity Disorder (ADHD)

A model example of how to interpret effect sizes against external benchmarks is provided by Bernanke and colleagues (Bernanke et al., 2022), who contextualized their findings on structural brain differences in ADHD by directly comparing effect sizes from ABCD with those from a prior meta-analysis. Rather than simply reporting statistical significance, they visualized their effect sizes alongside published benchmarks from the ENIGMA ADHD Working Group, allowing for more informed judgment about the magnitude and relevance of their findings.

In their Figure 2, Bernanke et al. plotted effect sizes for regions of interest (ROI) in the ABCD dataset relative to those reported in the ENIGMA meta-analysis. In that meta-analysis, Cohen’s *d* values ranged from −0.21 to −0.10 for cortical thickness measures comparing youth with ADHD to typically developing controls. In contrast, Bernanke et al.’s ABCD analysis (949 ADHD participants vs. 9787 controls) identified 11 statistically significant group differences across 79 structural brain measures, with effect sizes ranging only from *d* = −0.11 to −0.06. Notably, all cortical thickness effects in ABCD were smaller than those in the ENIGMA findings.

Their conclusion that children with ADHD “differed only modestly on structural brain measures” appears well justified in light of the effect sizes, though arguably even the largest effects (e.g., *d* = −0.11) may fall below thresholds for practical or clinical relevance. By juxtaposing their effects with meta-analytic estimates, and plotting them side by side, the authors enabled readers to assess the results not just for significance, but for magnitude and meaning.

Indeed, in his commentary on the paper, Halperin (2022) observes that these findings suggest continued searches for smaller neuroanatomical differences in ADHD may not be fruitful. This judgment is debatable, but importantly, it is rooted in a comparative evaluation of effect sizes. Such interpretation reflects good practice in large-sample studies like ABCD, where precise estimation allows us to focus on whether effects are truly meaningful. Still, some critical steps were missing from the analysis—particularly the *a priori* specification of a smallest effect size of interest (SESOI). For example, if a structural brain difference must exceed *d* = 0.20 to meaningfully differentiate diagnostic groups (e.g., a benchmark based on other neurodevelopmental conditions), then all of the observed effects in this study would fall short. An equivalence test could formally evaluate whether the true effects are statistically smaller than this SESOI, thus strengthening claims that group differences are negligible in practical terms. While the SESOI may vary by brain region or functional consequence, incorporating such thresholds is essential for transitioning from statistical to substantive interpretation.

To motivate this idea, consider Parkinson’s disease: clinical symptoms do not typically emerge until approximately 60% of dopaminergic neurons in the *substantia nigra* are lost (Dauer and Przedborski, 2003, Fearnley and Lees, 1991). This striking example illustrates that biological systems can often sustain substantial structural changes before producing observable effects. In the context of ADHD, we do not yet know what degree of volumetric difference constitutes a meaningful biological signal—would a 10% difference in volume be clinically significant? Would this threshold vary by brain region or by functional domain? These are empirical questions, but they emphasize the value of defining SESOIs that are anchored in theoretical models, clinical benchmarks, or known biological thresholds. Making these thresholds explicit, ideally before analyzing the data, strengthens the interpretive clarity of large-sample studies.

Overall, Bernanke et al. demonstrate several good practices — reporting exact effect sizes, visualizing them against known benchmarks, and avoiding overinterpretation of statistically significant but small effects. It would have been beneficial to include confidence intervals of the effect size estimates. In addition, future studies could build on this approach by integrating SESOI and equivalence testing frameworks to more rigorously assess whether observed differences are practically or clinically important.

## 6 Recommendations: Define, compare, test, visualize

We conclude with four actionable recommendations for interpreting effect sizes in large-scale studies such as ABCD. These practices are designed to move beyond the limitations of null hypothesis significance testing and toward a more transparent, cumulative, and practically relevant science.

**Define.** Interpretation begins by defining meaningful effects upfront in the framing phase of the investigation. What would be defined as meaningful and why? We gave an example of the definition of the SESOI. This is the smallest magnitude of effect that would be considered theoretically or practically meaningful. The SESOI can be based on clinical thresholds, policy implications, theoretical expectations, or empirical benchmarks (e.g., from prior meta-analyses). Explicitly defining the SESOI before examining the data helps clarify the aims of the research and prevents arbitrary interpretation of small, but statistically significant, effects. In Example 3, for instance, if one defined *d* = 0.20 as a meaningful group difference in brain volume, the observed ADHD effects could be formally evaluated against that benchmark.

**Compare.** Once an effect size is estimated, it should be interpreted relative to meaningful standards. These include prior findings (e.g., meta-analytic estimates), theoretical predictions, or normative distributions of effect sizes in the field. Comparative interpretation situates new findings in context, preventing misinterpretation of small effects as important simply because they are statistically significant. Bernanke et al.’s ADHD study (Example 3) exemplified this approach by comparing ABCD-derived effect sizes to those from the ENIGMA consortium, revealing that the ABCD effects were consistently smaller.

**Test.** Equipped with a defined SESOI and contextual comparisons, researchers can then test whether the observed effect is meaningfully different from zero—or from the SESOI itself. Equivalence testing, for example, allows researchers to formally evaluate whether an effect is smaller than the smallest size of interest, supporting conclusions that the effect is negligible. Confidence intervals, too, provide critical information about the precision and plausible range of an effect. In large-sample studies like ABCD, where even trivial effects can be statistically significant, testing whether effects are practically meaningful is essential.

**Visualize.** Visualization complements these steps by making complex statistical information more accessible and interpretable. Estimation graphics, confidence interval plots, and comparisons to SESOI thresholds can help readers intuitively grasp both the size and precision of an effect. Visualization is especially helpful in large datasets, where the magnitude of an effect may not be obvious from a *p*-value alone. As seen in our examples, graphical representations can effectively highlight trivial effects that are statistically significant, convey overlap between groups, and contextualize results relative to meaningful benchmarks.

**Summary.** In large-scale studies like ABCD, where nearly all effects can become statistically significant, interpreting results requires a principled shift in focus. By explicitly **defining** what constitutes a meaningful effect, **comparing** observed effects to relevant standards, **testing** whether effects meet thresholds of practical importance, and **visualizing** results to aid interpretation and communication, researchers can draw more meaningful, transparent, and scientifically valuable conclusions.

## CRediT authorship contribution statement

**Anthony Steven Dick:** Methodology, Visualization, Supervision, Data curation, Writing – original draft, Validation, Software, Investigation, Formal analysis, Resources, Writing – review & editing, Project administration, Funding acquisition, Conceptualization. **Jonathan S. Comer:** Writing – review & editing, Conceptualization. **Mohammadreza Bayat:** Writing – review & editing. **Marilyn Curtis:** Writing – review & editing. **Timothy Hayes:** Writing – review & editing. **Shannon M. Pruden:** Writing – review & editing. **Samuel W. Hawes:** Writing – review & editing. **Raul Gonzalez:** Funding acquisition, Writing – review & editing. **Angela R. Laird:** Writing – review & editing, Funding acquisition. **Paulo A. Graziano:** Funding acquisition, Writing – review & editing.

## Declaration of competing interest

The authors declare that they have no known competing financial interests or personal relationships that could have appeared to influence the work reported in this paper.

## Appendix A Supporting information

Code for all analyses and figures are supplied at https://github.com/anthonystevendick/Meaningful-Effects-Redux.

## Footnotes

1 The data were from a large sample of 19-year-olds in the United States: *n*= 5092 for males and *n*= 5510 for females. We take the summary statistics from a sample this size to be a reasonable estimate of the population, under standard sampling assumptions (independent and identically distributed), but recognize that there is error in this estimate. We use these data here for illustration only.

2 Note, this equivalence is specific to a regression with only one predictor variable.
